# Simple Prognostic Criteria can Definitively Identify Patients who Develop Severe Versus Non-Severe Dengue Disease, or Have Other Febrile Illnesses

**DOI:** 10.4021/jocmr694w

**Published:** 2012-01-17

**Authors:** Andrew K.I. Falconar, Claudia M.E. Romero-Vivas

**Affiliations:** a Laboratorio de Enfermedades Tropicales, Departamento de Medicina, Fundacion Universidad del Norte Km5 Antigua Via a Puerto Colombia, Barranquilla, Colombia, South America

## Abstract

**Background:**

Severe dengue disease (SDD) (DHF/DSS: dengue hemorrhagic fever/dengue shock syndrome) results from either primary or secondary dengue virus (DENV) infections, which occur 4 - 6 days after the onset of fever. As yet, there are no definitive clinical or hematological criteria that can specifically identify SDD patients during the early acute febrile-phase of disease (day 0 - 3: < 72 hours). This study was performed during a SDD (DHF/DSS) epidemic to: 1) identify the DENV serotypes that caused SDD during primary or secondary DENV infections; 2) identify simple clinical and hematological criteria that could significantly discriminate between patients who subsequently developed SDD versus non-SDD (N-SDD), or had a non-DENV fever of unknown origin (FUO) during day 0 - 3 of fever; 3) assess whether DENV serotype co-infections resulted in SDD.

**Methods:**

First serum samples, with clinical and hematological criteria, were collected from 100 patients during the early acute febrile-phase (day 0 - 3: < 72 hours), assessed for DENV or FUO infections by IgM- and IgG-capture ELISAs on paired serum samples and by DENV isolations, and subsequently graded as SDD, N-SDD or FUO patients.

**Results:**

In this study: 1) Thirty-three patients had DENV infections, predominantly secondary DENV-2 infections, including each SDD (DHF/DSS) case; 2) Secondary DENV-2/-3 and DENV-2/-4 serotype co-infections however resulted in N-SDD; 3) Each patient who subsequently developed SDD, but none of the others, displayed three clinical criteria: abdominal pain, conjunctival injection and veni-puncture bleeding, therefore each of these criteria provided definitively significant prognostic (P < 0.001) values; 4) Petechia, positive tourniquet tests and hepatomegaly, and neutrophilia or leukopenia also significantly identified those who: a) subsequently developed SDD versus N-SDD, or had a FUO; b) subsequently developed SDD versus N-SDD; c) subsequently developed N-SDD versus FUOs, respectively.

**Conclusions:**

This is the first report of simple definitively prognostic criteria for SDD patients, including the first assessment and confirmation of conjunctival injection. The three definitive clinical criteria used alone, or supported by the other four criteria, could be essential for specifically identifying those patients needing prompt hospital-based therapies to lessen or avert SDD, without unnecessary hospitalization of the other patients.

**Keywords:**

Dengue virus; Severe dengue; Dengue fever; Diagnostic; Criteria; Hemorrhage; Shock

## Introduction

There has been an alarming increase in the incidence of the vector-borne viral disease dengue fever (DF), and its severe life-threatening forms, dengue hemorrhagic fever/dengue shock syndrome (DHF/DSS) throughout tropical and subtropical regions of the world [1]. Peak human infection rates occur after periods of increased rainfall due to increased replication of the principal dengue virus (DENV) mosquito vector species, *Aedes aegypti* [2]. Under-reporting or misdiagnosis of DENV infections often occurs due to both the lack of adequately equipped laboratories in many endemic areas, and the wide-range and over-lapping spectra of clinical symptoms of infections caused by DENV and other infectious agents [3]. While most DENV-infected patients display an undifferentiated fever (DF: classical dengue fever), DENV infections may also result in thrombocytopenia, severe hemorrhage, and fatal shock [4], with some patients also developing hepatitis, myo-carditis, renal disease, respiratory distress, encephalopathy or encephalitis [5]. Thus, new classifications were introduced to differentiate between patients with non-severe dengue disease (N-SDD) and those who develop warning signs (i.e. those requiring observation and medical intervention) and severe dengue disease (SDD) (severe plasma leakage (DHF), leading to shock (DSS), fluid accumulation with respiratory distress, severe bleeding, and/or severe organ disease) [6].

Serological assays, such as IgM and IgG capture ELISAs are routinely employed for the diagnosis of DENV infections but require paired patients’ S1 and S2 serum samples collected 2 - 14 days apart to confirm a greater than four-fold increase in DENV-specific IgG and/or IgM titers [4]. These assays are, however, usually negative within the first four days after the onset of fever in patients experiencing primary DENV infections, with approximately 50% of them being IgM positive on day 5 [7]. Up to 32% of patients with secondary DENV infections may never generate detectable DENV-specific IgM antibodies [8]. Alternatively, DENV-infected patients may be identified by performing reverse transcription polymerase chain-reactions (RT-PCRs), or by DENV isolations using mosquito cell-lines, coupled with their detection using either RT-PCR or DENV antigen-specific monoclonal antibodies (MAbs) [4,6].

Since only a small proportion of patients with secondary DENV infections result in SDD (DHF/DSS) [9], many studies have been performed to identify clinical and hematological criteria that may discriminate between patients who have or will subsequently develop SDD (DHF/DSS) versus N-SDD (DF). In one study, aimed to identify early acute febrile phase prognostic markers in Colombia, there was a significant association between vomiting, gingival hemorrhage, microscopic hematuria and increased hematocrit, but not hepatomegaly, and subsequent SDD (DHF/DSS) development [10]. In another Colombian study, age was identified as a major factor for the development of SDD but, whilst ascites, pleural effusion and bleeding, but not abdominal pain or vomiting, were also risk factors for the development of respiratory distress and hypotension (SDD), the earliest day on which they were observed was not provided [11]. In contrast, a study of adults and older children (mean 34.7 ± 15.1 (range 8 - 86 years old)) in Mexico, did not identify age as a SDD risk factor, but ascites, thrombocytopenia (40,000 - 60,000/μl), persistent vomiting and some symptoms of bleeding (e.g. gingivorrhagia and hematemesis), but not petechias, ecchymoses or a positive tourniquet test, were confirmed to be warning signs of imminent SDD [12]. In another study performed in Brazil, a positive tourniquet test, hematemesis and ecchymoses were found in SDD patients, but there was no correlation between thrombocytopenia on initial presentation (hospital admission) and symptoms of bleeding [13]. Early adult DHF/DSS prognostic algorithms assessed in Singapore, either relied on real-time RT-PCR assays to assess DENV titers, which are too complicated and expensive to routinely perform [14], or only had a low (48.1%) predictive accuracy, and only prevented 43.9% of patients who only developed N-SDD (DF) from being unnecessarily hospitalized [15]. In a more recent study, using clinical laboratory data obtained from Thai patients less than 72 hours after the onset of fever in multivariable logistic regression models, age with minimum platelet and white blood cell counts, and maximum hematocrit and neutrophil percentages, aspartate aminotransferase (AST) concentrations and tourniquet test petechia numbers (> 20/inch^2^), at a sensitivity of 79.6%, could differentiate between patients who subsequently developed SDD (DHF/DSS) versus N-SDD (DF) [16]. In a more recent study also performed on data collected in Thailand, two different classification and regression tree (CART) analyses using algorithms that combined: 1) reduced white blood cell numbers, monocyte percentages and platelet numbers; and 2) increased hematocrits (CART #1) or, a) age (> 6.75 years); b) reduced white blood cell numbers, neutrophil percentages, and platelet numbers; and c) increased aspartate aminotransferase (AST) concentrations (CART #2) were tested for their abilities to identify patients who subsequently developed SDD (DSS or severe plasma leakage, respectively) [17]. While these CART analyses could achieve a sensitivity of 97% in identifying the DENV-infected patients who subsequently developed DSS, they only correctly excluded 48% of those who subsequently developed N-SDD (DF) [17].

Thus, because of the differences in the clinical and hematological findings observed in DENV infected patients who subsequently developed SDD (DHF/DSS) in studies conducted in the Americas and South-east Asia, and between studies conducted in the same countries, there is an urgent need to further evaluate criteria in different SDD (DHF/DSS) endemic sites throughout the world. Simple clinical or hematological criteria that can identify such patients in poor SDD (DHF/DSS) endemic areas, that have poorly equipped laboratories, would be ideal so that prompt hospital-based supportive therapy can be provided to these patients to avert or reduce SDD.

In most of these previously described clinical studies [10-17], the abilities of each DENV serotype to cause SDD (DHF/DSS) in either primary or secondary DENV infections were not assessed. DHF/DSS epidemics began in the Americas after the introduction of virulent DENV-2 (American/Asian genotype) strains from Thailand that subsequently displaced more weakly pathogenic DENV-2 (American genotype) strains [18]. The DENV-3 Sri Lankan genotype IIIb strain was later introduced and which also caused DHF/DSS throughout the Americas [1], but virulent (DHF/DSS-associated) DENV-3 Asian I and V genotype strains were also isolated in South America [19,20]. While most cases of SDD (DHF/DSS) were previously associated with secondary DENV infections [9], many patients with primary infections, particularly with DENV-1 strains, also developed DHF/DSS [21]. More recently, there have been many reports in which patients with primary DENV infections developed SDD (DHF/DSS) in: 1) Thailand (primary DHF: 13.2%) [22], and which were predominantly caused by DENV-1 and DENV-3 serotypes (primary DHF: DENV-1: 20%, DENV-3: 21%) [23] (primary DHF: DENV-1: 28%, DENV-3: 33%) [24]; 2) Viet Nam, mainly caused by DENV-1 compared with DENV-2 (primary DHF: 18%) [25]; 3) Colombia, mainly by DENV-3, but less so by DENV-2 [26]; 4) Brazil, predominantly by DENV-3 (primary DHF: 42.7%) [27] (primary DHF: 55.2%) [28]. It is therefore very important to determine whether SDD in patients resulted from either primary or secondary DENV infections, and to identify the DENV serotypes responsible in each case. We previously defined the terms ‘classical’ and ‘non-classical’ acute primary and secondary DENV antibody responses since the latter group included: 1) high S1 IgG acute primary (*P); 2) high S1 IgM acute secondary (*S); 3) high S1 IgM and low S2 IgG acute secondary (*LS) DENV infections [8]. As a result, the three previously published IgM:IgG (> 1.2 and < 1.2, > 1.4 and < 1.4 and > 1.78 and < 1.78) discriminatory optical density (DOD) ratios could not reliably classify them using IgM:IgG OD ratios obtained from their early acute-phase (day 0 - 3: < 72 hour) S1 serum samples [8]. Higher > 2.60 and < 2.60 DOD ratios were therefore derived [8].

We also reported the presence of DENV-1 and the virulent DENV-2 American/Asian genotype strain (PTCOL96) (GenBank AF 163096.1) in the Magdalena River Valley of Colombia [29,30]. DENV-1, DENV-2 and DENV-4 were subsequently shown to co-circulate in Barranquilla, which is the principal sea port of Colombia, during 2000 and 2001 [8]. These DENV serotypes, of which DENV-4 was dominant, were mainly identified in secondary DENV infections, but only caused N-SDD (DF) [8]. Cases of SDD (DHF/DSS) were, however, subsequently reported in Barranquilla.

In this study, we therefore tested: 1) whether SDD resulted from either primary or secondary DENV infections; 2) which DENV serotypes generated SDD in these patients; 3) whether the patients who subsequently developed SDD (DHF/DSS) could be significantly differentiated from those who developed N-SDD (DF) or had a fever of unknown origin (FUO), during the early acute-phase of disease (day 0 - 3: < 72 hours), using a panel of simple clinical and hematological criteria; 4) whether patients infected with the virulent (SDD-associated) DENV serotype strain would result in more severe clinically-graded disease when they were also co-infected with another DENV serotype; 5) whether any of these simple clinical or hematological criteria could also discriminate between DENV-infected patients, and those who had an FUO.

## Materials and Methods

### Ethics statement

The Universidad del Norte Ethics Committee, following Colombian national guidelines, approved this study involving human patients. Written consent was obtained from all of the patients, or in the case of children, from their parents before any patients’ samples or details were collected.

### Study area

Barranquilla, which has a population of 1.2 million people, is the principal sea-port of Colombia, and lies on the Caribbean coast at 14 meters above sea level. The average annual temperature is 28 ^o^C, and two wet seasons occur throughout the year. This city is endemic for DENV transmission, with three of the four DENV serotypes previously found to co-circulate [8]. This study was performed during a 2007 epidemic of SDD in the Por Fin district of Barranquilla. After written consent was obtained, first (S1) blood samples were collected from a total of 100 patients who presented at clinics on either the day of onset of febrile illness (day 0), or day 1 to 3 (< 72 hours) after the onset of febrile illness. Full clinical details were obtained from each patient, hematological studies were performed on the blood samples, and their serum samples were separated and stored at -85 ^o^C. Each of these patients were subsequently followed up to record whether they required hospitalization, and 2 to 10 days after collecting their S1 samples, second (S2) blood samples were obtained from each of them. The hospitalized patients were further clinically classified on day 4 - 6 after the onset of symptoms as having SDD caused by DHF grades I, II, III and IV, without or with severe organ pathologies, according to strict WHO guidelines [4,6]. As such, DHF I was determined by DF symptoms with thrombocytopenia (< 100,000/μl), a positive tourniquet test and evidence of plasma leakage (> 20% increased hematocrit), DHF II was determined by DHF I criteria together with spontaneous bleeding, DHF III was determined by DHF I or II criteria with evidence of circulatory failure (narrowed systolic pressure by < 20 mmHg (< 2.7 kPa), and DHF IV was determined by the criteria used for DHF I and II, together with evidence of profound shock (undetectable blood or pulse pressures) on day 4 - 6 after the onset of symptoms [4,6].

### Growth of DENV and identification of DENV serotypes

The use of DENV strains of each serotype for the ELISAs and the immuno-fluorescent assays (IFAs) were described [8]. Briefly, DENV-1 (Nauru Island strain), DENV-2 (New Guinea-C prototype strain), DENV-3 (PR 1340 strain) and DENV-4 (Dominica strain) were grown in 70% confluent C6/36 (*Aedes albopictus*) cell monolayers, maintained in 40 ml of Leibovitz (L-15) medium containing 10% (v/v) tryptose phosphate broth, 10% (v/v) fetal bovine serum and antibiotics in 80 cm^2^ culture flasks. After incubation at 28 ^o^C for 4 days, the supernatants were collected and replaced with fresh media and incubated for another 4 days to obtain a second harvest. These supernatants were made to 50 mM Tris/HCl pH 7.2, clarified by centrifugation at 200 × g, and aliquots were stored at -85 ^o^C. The cell pellets were re-suspended in a minimal volume of PBS and added to each well of 12-well polytetrafluoroethylene (PTFE) coated immuno-fluorescent slides (Hendley, UK), air-dried, fixed with cold (-20 oC) acetone, again air-dried and stored at -40 ^o^C.

The methods used for DENV isolation by cell-culture and their subsequent detection using serotype-specific monoclonal antibodies (MAbs) were described [8]. Briefly, 100 μl volumes of the patients’ S1 serum samples were 0.2 μm filter-sterilized onto 70% confluent C6/36 (Aedes albopictus) cell monolayers maintained in 2.5 ml cell culture medium in 25 cm^2^ flasks. After incubation for 2 hours at 28 ^o^C, the supernatants were collected and any detached cells were re-introduced into the flasks after centrifugation, discarding the supernatants, and re-suspending the cell-pellets in 10 ml of fresh media. After incubation at 28 oC for 7 - 10 days, the supernatants were harvested, made to 50 mM Tris/HCl pH 7.2, clarified by centrifugation and stored at -85 oC. The cell pellets were re-suspended in a minimal volume of PBS, added to IFA slides, dried, fixed, re-dried and stored at -40 ^o^C.

### Immunofluorescent assays (IFAs)

Monoclonal antibodies (MAbs), specific for the envelope (E) glycoproteins of all flaviviruses (MAb 4G2), DENV-1 (MAb 15F3), DENV-2 (MAb 3H5), DENV-3 (MAb 5D4) or DENV-4 (MAb 1H10) (ATCC, USA) were diluted to 1/100 in PBS containing 2% milk powder (Marvel, Cadbury’s, UK) and reacted with the fixed C6/36 cells on the IFA slides for 2 hours at 28 ^o^C. These slides were then washed three times with PBS and gently blotted before 10 μl of a 1/500 dilution of FITC-labeled goat anti-mouse IgG (H&L) was added, and they were incubated at 28 ^o^C for 1 hour. After washing three times with PBS, the slides were briefly dipped in distilled water, gently blotted, mounted with 90% (v/v) glycerol/PBS pH 8.3, and viewed under immuno-fluorescent microscopy using the appropriate FITC excitation and barrier filters.

### MAC and GAC ELISAs

The IgM- (MAC) and IgG- (GAG) capture ELISAs were described [8]. For these assays, 20 μg/ml of goat anti-human IgM (μ-chain specific) and anti human IgG (γ-chain specific) (109-005-129 and 109-005-098: Jackson ImmunoResearch, USA) were added at 50 μg/well in 1.6%/2.93% (w/v) sodium carbonate/bicarbonate buffer (pH 9.8) to high-binding ELISA plates (Immulon 4, Dynatech, USA). These plates were then incubated overnight at 4 ^o^C. After washing with PBS containing 0.05% (v/v) Tween 20 (P 1379; Sigma) (PBS/T), they were blocked using 120 μl/well of PBS containing 1% (w/v) gelatin (G 6650; Sigma) at 37 ^o^C for 2 hours. Serial four-fold dilutions of patients’ sera, or control positive and negative patients’ sera, from 1/40 were prepared in triplicate in PBS/T with 0.25% gelatin (PBS/T/G) (140 μl volumes) in low binding ELISA plates (Immulon 1; Dynatech, USA). After washing the blocked ELISA plate with PBS/T, 50 μl volumes of the diluted sera were rapidly transferred to this plate working up the concentration gradients for IgM and IgG capture. After incubation at 37 ^o^C for 2 hours, these plates were washed with PBS/T and a 1/3 dilution of the C6/36 cell-culture supernatants infected with each of the four DENV serotypes, prepared in PBS/T/G (50 μl/well), were added and incubated at 37 ^o^C to capture the DENVs. After washing again, a 1/60,000 dilution of purified MAb 2C5.1 prepared in PBS/T/G was allowed to react with the captured DENV at 37 ^o^C for 2 hours. After washing, the bound MAb was detected by sequential reaction steps with a peroxidase-labeled goat anti-mouse IgG (H&L) second antibody (115-035-062; Jackson ImmunoResearch, USA), and 0.012% (w/v) ο-phenylenediamine dihydrochloride (P 1526; Sigma) substrate in 0.09% (w/v) citrate-phosphate buffer (pH 5.0) containing 0.09% (v/v) H_2_O_2_ (50 μl/well). After incubation for 10 minutes, the reactions were stopped by the addition 2M H_2_SO_4_ (25 μl/well) and absorbances were determined at dual 490 and 630 nm wavelengths (MRX; Dynex). Average optical density (OD) values for each dilution of positive and negative patients’ sera [8] were used to draw graphs and establish absorbance max/2 values for their 1/log_10_ 50% end-point IgM- and IgG-capture (MAC and GAC) ELISA titers (1/log_10_t_50_). Patients were reported to have current flavivirus infections when they showed greater than 4-fold increases in DENV-specific IgM or IgG 50% end-point titers titers (1/log_10_t_50_) between their S1 and S2 serum samples. Primary or secondary DENV-infections were identified by applying > 2.6 and < 2.6 or >1.8 and <1.8 discriminatory OD (DOD) ratios to their S1 and S2 sample IgM:IgG OD ratios obtained at 1/100 dilutions, respectively [8].

### Statistical Analyses

The data were analyzed using EPIINFO 6.0 and comparisons between the clinical and hematological values of each group of patients were performed using Fisher’s exact test, from which two-tailed p values of < 0.05 were considered statistically significant. Comparisons of the mean ages of the patients who subsequently developed SDD or N-SDD, or had a FUO were performed using the t-test for independent samples using MedCalc statistical software Version 11.6.

## Results

### Patients

For this study, 100 paired blood samples, in which their first (S1) samples were obtained on day 0 to 3 (< 72 hours) after the onset of febrile disease, were collected at out-patients clinics during a SDD (DHF/DSS) epidemic. Full clinical and hematological parameters were assessed upon presentation, which included the new WHO criteria for probable DENV infections: nausea, vomiting, rash, aches and pains, positive tourniquet test or leukopenia, with or without the ‘warning signs’ of abdominal pain or tenderness, persistent vomiting, clinical fluid accumulation, mucosal bleeding, liver enlargement (> 2 cm), increased hematocrit (% PCV), with concurrently decreased platelet numbers [6]. All patients were then further assessed, when second (S2) blood samples were obtained from each of them 2 - 10 days later. Any hospitalized patients were diagnosed as having DHF grades I to IV or other symptoms of SDD, using strict WHO guidelines [4,6].

### Serological and virological results

Of the 100 patients samples collected in this study, most (67%: 67/100) had a fever of unknown origin (FUO), since they showed no reaction in the DENV IgM- and IgG-capture ELISAs using either their S1 or S2 serum samples. The others (33%: 33/100) were confirmed to have acute DENV infections by obtaining greater than 4-fold increase in their IgM- or IgG-capture ELISA titers (1/log_10_t_50_) between their S1 and S2 samples (Table 1). These patients ranged from 1 - 27 years old, with their mean age being 8.1 years, and a male: female ratio of 0.94. Thus, predominantly young children were infected during this SDD (DHF/DSS) epidemic.

**Table 1:** Viral and/or Serological Confirmation of DENV Infections in 33/100 Patients

**No**	**Age (y)**	**DOC^a^**		**Virus^b^**	**1/log_10_ ELISA titer (OD at 1/100 dilution)^c^**						**Clinical^d^ Classification**
					**S1**		**IgM/IgG OD ratio (P/S)**	**S2**		**IgM/IgG OD ratio (P/S)**	
		**S1**	**S2**		**IgM**	**IgG**		**IgM**	**IgG**		
1	19	0	6	-	-(0.4)	-(0.7)	0.6(S)	3.5(1.5)	5.6(1.6)	0.9(S)	N-SDD
2	14	1	9	D-2V	-(0.2)	-(0.5)	0.4(S)	4.1(1.6)	6.6(1.6)	1.0(S)	SDD(DHF I)
3	10	1	4	D-2V	-(0.5)	-(0.3)	1.7(S)	3.2(1.3)	5.6(1.6)	0.8(S)	N-SDD
4	6	1	5	D-2V	-(0.4)	-(0.2)	2.0(S)	3.3(1.5)	6.6(1.6)	0.9(S)	N-SDD
5	7	1	4	-	-(0.3)	-(0.7)	0.4(S)	2.3(1.0)	-(0.32)	3.1(*P)	N-SDD
6	6	1	3	D-2V	3.2(1.3)	-(0.3)	4.3(P)	3.2(1.4)	5.4(1.6)	0.9(*S)	N-SDD
7	11	1	10	D-2/-3V	-(0.6)	4.3(1.1)	0.6(S)	3.4(1.0)	6.3(1.6)	0.6(S)	N-SDD
8	1	1	10	-	-(0.7)	-(0.2)	3.5(P)	2.9(1.3)	-(0.3)	4.3(P)	N-SDD
9	8	2	4	D-2V	-(0.4)	4.2(1.2)	0.3(S)	2.9(1.0)	5.7(1.6)	0.6(S)	SDD(DHF III)
10	22	2	7	D-2V	-(0.2)	4.4(1.1)	0.2(S)	3.1(1.0)	6.7(1.6)	0.6(S)	SDD(DHF II)
11	7	2	8	D-2V	-(0.3)	-(0.1)	3(P)	3.7(1.5)	4.9(1.5)	1(*S)	N-SDD
12	9	2	6	-	2.1(0.9)	4.5(0.9)	1(S)	2.9(1.2)	6(1.6)	0.8(S)	N-SDD
13	2	2	6	-	-(0.5)	-(0.3)	1.7(S)	4.6(1.6)	3.5(1.4)	1.1(*LS)	N-SDD
14	18	2	8	D-2V	-(0.3)	5(1.2)	0.3(S)	3.9(1.3)	5.8(1.6)	0.8(S)	SDD(DHF II)
15	10	2	5	D-2V	-(0.3)	-(0.1)	3(P)	3.2(1.4)	4.9(1.5)	0.9(*S)	N-SDD
16	8	2	8	D-2V	-(0.4)	-(0.2)	2(S)	3.6(1.6)	5.7(1.6)	1(S)	N-SDD
17	10	2	5	-	-(0.8)	-(0.4)	2(S)	3.6(1.6)	6.6(1.6)	1(S)	N-SDD
18.0	6	2	9	D-2V	-(0.5)	-(0.3)	1.7(S)	3.5(1.6)	5.6(1.6)	1(S)	N-SDD
19	27	2	7	D-1V	-(0.4)	4.2(0.8)	0.5(S)	2.9(1.0)	5.7(1.6)	0.6(S)	N-SDD
20	11	2	8	-	-(0.6)	4.6(1.0)	0.6(S)	3.2(1.3)	6.1(1.6)	0.8(S)	N-SDD
21	6	2	4	D-2V	-(0.3)	-(0.1)	3(P)	2.6(1.1)	-(0.4)	2.8(P)	N-SDD
22	14	2	5	D-3V	-(0.3)	-(0.2)	1.5(S)	-(0.8)	5.2(1.6)	0.5(S)	N-SDD
23	9	2	8	D-2V	-(0.3)	4.7(1.1)	0.3(S)	-(0.6)	6.3(1.6)	0.4(S)	N-SDD
24	6	3	5	D-2V	-(0.6)	-(0.7)	0.9(S)	3.6(1.6)	5.3(1.6)	1(S)	N-SDD
25	11	3	9	D-2/-4V	-(0.3)	-(0.5)	0.6(S)	-(0.5)	5.9(1.5)	0.3(S)	N-SDD
26	11	3	9	-	-(0.4)	2.1(0.9)	0.4(S)	2.3(0.9)	6.1(1.6)	0.6(S)	N-SDD
27	11	3	13	-	-(0.4)	-(0.6)	0.7(S)	-(0.7)	6.8(1.6)	0.4(S)	N-SDD
28	7	3	6	D-2V	-(0.2)	-(0.6)	0.3(S)	3.0(1.1)	6.5(1.6)	0.7(S)	SDD(DHF III)
29	6	3	5	-	-(0.13)	4.5(1.4)	0.9(S)	3.5(1.3)	6.2(1.6)	0.8(S)	N-SDD
30	11	3	5	-	2.8(1.0)	-(0.6)	1.7(S)	3.7(1.3)	5.9(1.6)	0.8(S)	N-SDD
31	7	3	5	-	3.5(1.6)	5.3(1.6)	1(S)	3.9(1.6)	6.2(1.6)	1(S)	N-SDD
32	7	3	10	-	3.5(1.4)	5(1.6)	0.9(S)	4(1.6)	6(1.6)	1(S)	N-SDD
33	7	3	10	-	1.7(0.9)	4.6(1.2)	0.8(S)	3(1.5)	5.9(1.6)	0.9(S)	N-SDD

^a^DOC: day of S1 and S2 serum collection after the onset of fever. ^b^Dengue (-1 to -4) virus serotype isolated. ^c^Reciprocal log10 50% end-point IgM and IgG ELISA titers and optical densities (ODs) at 1/100 dilutions (in brackets) to identify primary (P) or secondary (S) DENV infections using ≥ 2.6 and < 2.6 and ≥ 1.8 and < 1.8 IgM:IgG OD ratios on their S1 and S2 samples, respectively, or high S1 IgG primary (*P), high S1 IgM secondary (*S) or low S2 IgG secondary (*LS) infections [8]. ^d^Clinical classifications as non-severe dengue disease (N-SDD) or severe dengue disease (SDD) with the DHF (I to IV) grade in brackets.

Most of these patients displayed ‘classical’ acute primary or secondary DENV antibody responses, which were, as previously found [8], most accurately identified in their S1 serum samples using the higher > 2.6 and < 2.6 IgM: IgG discriminatory optical density (DOD) ratios, than the three other IgM: IgG (> 1.2 and < 1.2, > 1.4 and < 1.4 and > 1.8 and < 1.8) DOD ratios previously published [8]. One patient (patient #5) was however erroneously identified as acute secondary DENV infections due to the low (0.4) IgM: IgG DOD ratio obtained using their S1 sample. Three other patients (patient #6, #11 and #15) were also erroneously identified as acute primary DENV infections due to their very high (4.3, 3.0 and 3.0) IgM: IgG DOD ratios obtained using their S1 samples. Thus, nearly all (91%: 30/33) of these cases were caused by secondary DENV infections, with one patient (patient #13) being classified as a low S2 IgG acute secondary infection (*LS), due to their lower S2 sample IgG than IgM ELISA titers (1/log10t50 = 3.5 versus 4.6). A low proportion (4/30: 13%) of patients with secondary DENV infections had DENV-specific IgM-negative S1 and S2 samples (patient # 22, #23, #25 and #27), but which was lower than previously found in Barranquilla (12/27: 32%) [8].

DENV-1, DENV-2 or DENV-3 were isolated from the S1 samples of 19/33 (58%) of these patients, but DENV-2 (90%: 17/19) was the dominant DENV serotype. Two patients (patient # 7 and # 25) were, however, co-infected with DENV-2/-3 and DENV-2/-4, respectively. All patients (patient # 2, #9, #10, #14 and #28) (5/33: 15%) that subsequently developed SDD (DHF grades I to III), based on WHO criteria [4], had secondary DENV infections caused by DENV-2, which was therefore a highly virulent strain implicated in causing this SDD epidemic. The age range of these patients was 7 - 22 years old, with a male:female ratio of 0.6, while the most severe clinically-graded disease (DHF III = DSS) occurred in the youngest of these patients (patient # 28: 7 years old; patient # 9: 8 years old). Another twelve patients (patient # 3, #4, #6, #7, #11, #15, #16, #18, #21, #23, #24 and #25), who were also young children (age range 6 - 11 years old), had secondary DENV infections caused by DENV-2, or co-infections caused by DENV-2 together with either DENV-3 (patient # 7) or DENV-4 (patient # 25), (both 11 years old), but subsequently only developed non-severe dengue disease (N-SDD: DF).

### Clinical and hematological criteria obtained from patients upon presentation

All of these 33 DENV-infected patients (Table 1) were tested on the day of presentation (day 0 - 3 after the onset of febrile disease) using 20 clinical criteria and 10 hematological criteria. While all of the results for the hematological criteria are shown, only the results for 12/20 of the clinical criteria are shown on Table 2. Since the average age of the patients who subsequently developed SDD (13.8 ± 6.4 years) was higher than those who developed N-SDD (8.8 ± 4.7 years), there was a low but significant difference between them (p = 0.047). The average age of the patients with a FUO was however even higher (18.3 ± 15.8 years) and therefore a highly significant difference was obtained between these patients and those who developed N-SDD (p < 0.001). Amongst the other clinical criteria, hepatomegaly (< 2 cm) was observed in only 40% of the patients during the acute febrile phase of disease who subsequently developed SDD (DHF/DSS), but none of the other patients. Thus, this clinical criterion, observed in only some of these patients, was useful to significantly discriminate between the patients who subsequently developed SDD, and those who developed N-SDD (p = 0.02), or had a FUO (p = 0.003).

**Table 2:** Early Acute Phase- (< 72 Hour-) Clinical and Hematological Criteria of Patients Who Subsequently Developed Severe Dengue Disease (SDD) or Non-Severe Dengue Disease (N-SDD), or Had Fever of Unknown Origin (FUO)

**Clinical/ hematological criteria**	**SDD (n = 5)**	**N-SDD (n = 28 )**	**FUO (n = 67)**	**p-value**		
				**SDD vs N-SDD**	**SDD vs FUO**	**N-SDD vs FUO**
Mean age (years) sd (range)	13.8 ± 6.4 (7 - 22)	8.8 ± 4.7 (1 - 19)	18.3 ± 15.8 (0.5 - 59)	0.047	ns	< 0.001
Abdominal pain (%)	100.0	0.0	0.0	< 0.001	< 0.001	ns
Eye pain (%)	80.0	76.2	56.7	ns	ns	ns
Joint pain (arthralgia) (%)	100.0	76.2	73.1	ns	ns	ns
Hepatomegaly > 2cm (%)	40.0	0.0	0.0	0.02	0.0030	ns
Jaundice (%)	0.0	0.0	20.9	ns	ns	ns
Vomiting (persistent) (%)	80.0	47.6	55.2	ns	ns	ns
Conjunctival injection (%)	100.0	0.0	0.0	< 0.001	< 0.001	ns
Gum bleeding (%)	20.0	9.5	4.5	ns	ns	ns
Petechia (%)	100.0	38.1	24.6	0.02	0.0010	ns
Tourniquet test positive (%)a	100.0	28.6	20.8	ns	< 0.001	ns
Venipuncture bleeding (%)	100.0	0.0	0.0	< 0.001	< 0.001	ns
Mean leukocytes (x103/ µl) sd (range)	4.3 ± 1.0 (3.1 - 5.8)	5.0 ± 1.8 (1.7 - 8.0)	8.0 ± 5.4 (7.2 - 35.3)	-	-	-
Leukopenia (%)b	60.0	62.0	17.8	ns	ns	0.0010
Leukocytosis (%)c	0.0	0.0	28.6	ns	ns	ns
Mean lymphocytes (%) sd (range)	11.8 ± 6.3 (4.3 - 18.6)	33.0 ± 12.6 (12.1 - 58.3)	30.4 ± 17.9 (6.6 - 35.4)	-	-	-
Lymphocytosis (%)	0.0	14.0	9.5	ns	ns	ns
Lymphopenia (%)	100.0	66.7	51.4	ns	ns	ns
Mean monocytes (%) sd (range)	4.6 ± 2.8 (1.5 - 8.5)	9.8 ± 4.6 (0.9 - 22.8)	8.3 ± 4.7 (0.5 - 28.6)	-	-	-
Monocytosis (%)	40.0	81.0	59.2	ns	ns	ns
Monocytopenia (%)	0.0	4.8	5.6	ns	ns	ns
Mean neutrophils (%) sd (range)	83.1 ± 7.8 (76.6 - 89.9)	57.4 ± 15.7 (27.9 - 85.5)	57.7 ± 20.7 (21.1 - 95.5)	-	-	-
Neutropenia (%)d	0.0	28.6	9.3	ns	ns	ns
Neutrophilia (%)e	100.0	23.8	50.7	0.0030	ns	ns
Mean platelets (x103/ µl) sd (range)	136.6 ± 43.1 (73.0 - 202.0)	226.4 ± 157.8 (134.0 - 393.0)	253.2 ± 114.0 (128.0 - 527.0)	-	-	-
Thrombocytopenia (%)f	20.0	0.0	0.0	ns	ns	ns
Mean hematocrit (%) sd (range)	35.6 + 5.2 (28.0 - 41.3)	36.0 ± 3.3 (27.8 - 40.9)	36.1 ± 4.8 (33.7 - 38.6)	-	-	-
Haematocrit > 20% (%)	0.0	0.0	0.0	ns	ns	ns

Clinical or hematological criteria percentages, numbers, standard deviations (sd: +) and ranges (in brackets), with comparative p-values or non-significant differences (ns). WHO values for DENV infections are: ^a^positive tourniquet test: >20 petechia/ inch^2^; ^b^leukopenia: < 5,000/μl; ^c^leukocytosis: > 10,000/μl; ^d^neutropenia: < 40%; ^e^neutrophilia: > 70%, and ^f^thrombocytopenia: < 100,000/μl [4, 6].

Eight clinical criteria (abdominal pain, chills, conjunctival injection, headache, joint pain (arthralgia), petechia, positive tourniquet test, and veni-puncture bleeding) and two hematological criteria (lymphopenia and neutrophilia) were displayed by all five patients who subsequently developed SDD (DHF/DSS) (Table 2). Only three of these clinical criteria (abdominal pain, conjunctival injection, and veni-puncture bleeding) were not displayed by any of the patients who subsequently developed N-SDD or had a FUO, and therefore each of them yielded definitively significant discriminatory (P < 0.001) values. Petechia and a positive tourniquet test were also displayed by a low percentage of patients who subsequently developed N-SDD (petechia: 38.1%; positive tourniquet test: 28.6%), or had a FUO (petechia: 24.6%; positive tourniquet test: 20.8%), but both also gave highly significant discriminatory P = 0.02 and 0.03 (petechia) and 0.005 and < 0.001 (positive tourniquet test) values for the SDD versus N-SDD or FUO patients, respectively.

Each of the five patients who subsequently developed SDD also had neutrophilia, however this also occurred in 23.8% and 50.7% of the patients who subsequently developed N-SDD or had a FUO, respectively. Statistical significance (P = 0.03) was therefore only observed between the patients who subsequently developed SDD versus N-SDD.

Our inability to find a significant difference between thrombocytopenia or increased hematocrits (≥ 20%) in the patients, during the early acute phase of disease (< 72 hours), who subsequently developed SDD versus N-SDD, or had a FUO was due to both criteria only being significantly displayed by these patients on day 4-6 after the onset of fever, when SDD (DHF I to III) occurred, in addition to reduced (< 20 mmHg) blood pressures in patient #9 and #28 who developed DHF III (DSS).

Despite only 60% and 62% of the patients who subsequently developed SDD and N-SDD displaying leukopenia, respectively, this criterion could significantly discriminate between those patients who subsequently developed N-SDD and those who had a FUO (N-SDD versus FUO: P = 0.001). Additional DENV-associated complications (e.g. acute hepatitis, acute respiratory distress syndrome or encephalopathy/encephalitis) were not observed in any of the DENV-infected patients in this study, despite being increasingly reported in Colombia [31-34] and elsewhere in the world [5].

## Discussion

We previously isolated DENV-1, -2 and -4 serotypes in Barranquilla during 2000 and 2001, with DENV-4 being the most common, but without any SDD (DHF/DSS) cases [8]. In this study, only secondary DENV-2 infections were implicated in the development of SDD (DHF/DSS). The DENV-2 strain previously found in Barranquilla [8] (Romero-Vivas et al., unpublished), and further up the Magdelena River [30], were of the virulent American/Asian genotype, which caused the SDD (DHF/DSS) epidemic in Por Fin. During this study, DENV-2 was most frequently isolated, but DENV-3 was subsequently imported and co-circulated with the other DENV serotypes. In another study conducted in North-east Colombia, near the Venezuelan border, secondary DENV-2 infections were mainly associated with SDD, but some SDD also resulted from primary DENV-2 infections, while DENV-3 induced SDD (DHF) occurred more frequently during primary DENV infections [26]. During 2001-2007, only strains of the DENV-3 IIIb genotype, were isolated in Colombia, and were different from those present in Brazil or Central American countries [35]. In another study, however, DENV-3 genotype I was identified in several Colombian states, while in three others DENV-3 genotype I and IIIb strains co-circulated, but the pathogenic capacities (SDD-associations) of these DENV-3 genotype I strains were not provided [19]. DENV-3 genotype V strains were also isolated in Colombia and Brazil, and caused a large SDD (DHF/DSS) epidemic in Brazil (Rio de Janairo) in 2002 [20]. As such, it is assumed that any DENV-3 isolates from Colombia are likely to be virulent, and may cause SDD in either primary or secondary DENV infections, as observed in both Colombia [26] and Brazil [27,28]. DENV-3 genotype determinations of the isolates from Atlantico (Barranquilla) have, however, not been obtained.

A low number of SDD (DHF) cases were also caused by secondary DENV-1 infections in North-east Colombia, while no SDD cases were caused by DENV-4 [26]. While these results confirmed the presence of a virulent DENV-1 genotype in this region of Colombia, only DENV-1 strains of the V/1 and V/2 genotype were widespread in Colombia, while one DENV-1 strain of genotype I had been isolated in the south of this country [36]. Further surveillance of the pathogenic capacities of DENV-1, DENV-2 and DENV-3, in Barranquilla is therefore urgently required.

Despite their importance, few other studies have been performed to identify clinical or hematological criteria that can be used during the early acute phase of disease to significantly discriminate between patients who subsequently developed either SDD versus N-SDD, or had a FUO. While we found three definitive clinical criteria (abdominal pain, conjunctival injection and veni-puncture bleeding), only one of them (abdominal pain) is included as a WHO ‘warning sign’ for potential SDD [6], while veni-puncture bleeding is not listed as a WHO ‘warning sign’, and conjunctival injection has not been assessed as a SDD prognostic criterion in any clinical study so far published.

In two studies conducted in Colombia, the incidences of abdominal pain (57%), petechia (57%), positive tourniquet test (35%), and hepatomegaly (33%) in children with SDD (DHF/DSS) [31], and abdominal pain (67.5%), petechia (60%) and veni-puncture bleeding (24.4%) in all SDD (DHF/DSS) patients [37] were lower than those found in our study. Age was also a SDD (DHF/DSS) risk-factor in other studies conducted in Colombia [11], Brazil [27] and Thailand [17], but not in another study conducted in Colombia [37] or in Mexico [12]. In our study, age only weakly discriminated between the patients who subsequently developed SDD and N-SDD (P = 0.047) but strongly between the N-SDD patients and those with a FUO (P < 0.001).

We did not find that fever, exanthema, thrombocytopenia (< 150,000), leukopenia (< 4,000) or vomiting were criteria to differentiate between patients who subsequently developed SDD versus N-SDD, as found in Brazil [39]. In another study, leukocyte count (< 4,500/μl) and thrombocyte count (< 90,000/μl), but not positive tourniquet test, were associated with spontaneous bleeding in patients from a DENV-endemic area [40]. We did not, however, observe a correlation between thrombocyte numbers and bleeding, as was also found in a study conducted in Brazil [13], since only one patient (patient #14) had thrombocytopenia (73,000/μl) upon presentation, while all of the patients who subsequently developed SDD each displayed conjunctival injection, petechia and veni-puncture bleeding. In another study, conducted in southern Colombia (Neiva), there was a correlation between thrombocytopenia (< 20,000/μl) on the day of hospitalization and the subsequent development of shock (DSS) [33]. However, we observed thrombocyte numbers of 152,000 and 267,000 in patient #9 and #28 on the day of presentation (day 2 and 3 respectively), which dropped to 49,000 and 23,000 on day 4 and 5 respectively after the onset of fever when they developed shock (DSS: DHF III).

We also did not identify any DENV-infected patients that displayed abnormal clinical manifestations, as have been reported elsewhere in Colombia (e.g. hepatitis or neurological (encephalopathy/encephalitis), renal, cardiac (myo-carditis), pancreatic, or pulmonary disease) [31-34], and elsewhere in the world [5].

While CART analyses achieved a sensitivity of 97% for identifying the DENV-infected patients who subsequently developed DSS using data collected < 72 hours after the onset of fever, they only correctly excluded 48% of those who subsequently developed N-SDD (DF) [17]. In contrast, we identified simple clinical and hematological criteria that could completely discriminate between patients, during the < 72 hour period, who subsequently developed SDD (DHF/DSS) versus N-SDD, or had a FUO. These three simple, definitive clinical criteria (abdominal pain, conjunctival injection and veni-puncture bleeding), together with petechia, a positive tourniquet test, and neutrophilia, that were also significantly displayed by each of these patients, with or without hepatomegaly, will be extremely valuable to definitively identify the patients who will subsequently develop SDD, amongst the much larger numbers who will subsequently develop N-SDD, or have a FUO. Importantly, these criteria can easily be assessed in poorly equipped clinics, as are present throughout our study area, so that prompt hospital-based support therapy can be performed to lessen or avert the onset of SDD (DHF/DSS). Biochemical tests to identify increased creatinine kinase (CK) and lactate dehydrogenase (LDH), and reduced serum albumin (SA) concentrations [38], and increased aspartate aminotransferase (AST) concentrations [17] should be assessed in these hospitalized patients, due to their additional SDD-prognostic values [17,38].

In a review of 15 studies that compared clinical and hematological criteria of patients with infection caused by DENV or FUOs, patients with DENV infections had significantly lower platelet, white blood cell and neutrophil numbers, and high incidences of petechia [41]. In a further study performed in Thailand, minimum leukocyte (< 500/μl) and thrombocyte (< 25,000/μl) numbers, and maximum neutrophil (> 75%) percentages, AST (> 100 U/dL) concentrations and petechial (> 20/inch^2^) numbers in tourniquet tests, yielded the greatest odds ratios between patients infected with DENV and those with a FUO [17]. The new WHO criteria for the specific identification of DENV infections now include nausea, vomiting, rash, aches and pains, positive tourniquet test, or leukopenia [6]. However, we found that leukopenia (< 5,000/μl) could only significantly differentiate between patients who developed N-SDD and those who had a FUO.

Co-infections with different DENV serotypes are rare, since the requirement to infect these patients during the relatively short (5 - 7 (range 3 - 14) day) incubation period [4]. Therefore, only a few of these DENV co-infections have been confirmed in Asia (DENV-2/-3 and DENV-3/-4) [42] and the Americas (DENV-2/-3 and DENV-3/-4) [43], but no information was provided about whether increased disease severity was observed in these patients. Although homologous DENV genetic recombination events have been extensively documented [44-46], there have not yet been any reports of heterologous DENV-serotype recombination events. The potential of such events to result in new chimeric strains with a possibly greater ability to effect antibody-enhanced replication (AER), resulting in antibody-enhanced disease (AED) [47], needs to be investigated.

Different human leukocyte antigens (HLAs) and non-HLA gene polymorphisms were identified as risk factors for developing SDD (DHF/DSS) in different countries [48]. While most studies identified different HLA class I molecules associated with both DHF/DSS risk and protection, HLA DR4, which occurs at a high frequency in Amerindian populations, provided protection against SDD (DHF/DSS) [49]. In a Mexican mestizo population, HLA-DQB1*0302, which also occurs at a high frequency in Amerindians, was, in contrast, positively associated with DHF/DSS risk and negatively associated with DF [50], while as yet unidentified genes provided SDD (DHF/DSS) resistance in the black populations from Cuba and Brazil [51,52]. Our study site has a mainly mestizo population, but also a high percentage of the black (African) phenotype. As such, the differences in clinical and hematological criteria identified in our study and those reported previously, particularly from South-east Asia [16,17], may also result from differences in the DENV genotypes co-circulating in Colombia and southern South-east Asian countries [53], and/or the patients’ genetics. All of the DHF/DSS patients identified in our study were of mestizo phenotype and were all successfully treated in hospital.

### Conclusions

Nine important findings came from this study, namely: 1) only secondary DENV infections with DENV-2 resulted in SDD (DHF/DSS); 2) secondary DENV co-infections with DENV-2/-3 or DENV-2/-4 did not result in SDD (DHF/DSS) in the few cases observed; 3) despite finding only low numbers of patients who developed SDD (DHF/DSS), we identified three definitive clinical criteria that were displayed by each of these patients during the early acute phase of disease (< 72 hours after the onset of fever), but not by any of the patients who developed N-SDD or had a FUO; 4) despite conjunctival injection not being previously assessed, we found that this was a definitive criterion for the development of SDD; 5) these clinical criteria where further significantly supported since each of these patients also displayed petechia and yielded a positive tourniquet test; 6) neutrophilia and hepatomegaly could discriminate between the patients who subsequently developed SDD and N-SDD, and hepatomegaly could also discriminate between the SDD and FUO patients; 7) age could only weakly discriminate between the SDD and N-SDD patients; 8) leukopenia could significantly discriminate between the DENV-infected patients and those who had a FUO; 9) thrombocytopenia and hemo-concentration, which are both *sine qua non* criteria for DHF, as well as reduced blood pressures (DSS), which occur on day 4 - 6 after the onset of fever [4,6], were not significantly displayed by the patients during the early acute-phase who subsequently developed SDD (DHF/DSS).

We believe that, despite studying only relatively low numbers of patients due to the present rarity of SDD in our study site, this is the first report in which clinical criteria were identified that, either alone or with further significant support by other clinical and hematological criteria, could definitively identify patients during the early acute phase of illness who subsequently developed SDD (DHF/DSS) versus N-SDD, or had a FUO. This is also the first report to assess and identify conjunctival injection as a definitive criterion for the identification of the patients who subsequently develop SDD. While relatively rare in our city, we believe that implementing such criteria to avert or lessen SDD symptoms using prompt appropriate therapies, will be important in this city, elsewhere in the Caribbean coast of Colombia, and possibly in the world. Further studies are therefore being conducted to test the prognostic accuracies of these criteria in other cities on the Caribbean coast of Colombia where SDD is also relative rare, as well as other cities in Colombia where SDD (DHF/DSS) cases are more common.
